# Perceptions and Management of Pregnancy‐Related Skin Changes: A Cross‐Sectional Study on Knowledge, Practices, and Use of Skincare Product

**DOI:** 10.1111/jocd.70132

**Published:** 2025-03-27

**Authors:** Safwan Aladwan, Reem Issa, Wala'a Al. Safadi, Lilian Alnsour, Lidia Kamal Al‐Halaseh

**Affiliations:** ^1^ Department of Cosmetic Science, Faculty of Allied Medical Sciences Al‐Ahliyya Amman University Amman Jordan; ^2^ Faculty of Pharmacy Middle East University Amman Jordan; ^3^ Biopharmaceutics and Clinical Pharmacy Department, Pharmacological and Diagnostics Research Centre, Faculty of Pharmacy Al‐Ahliyya Amman University Amman Jordan; ^4^ Department of Pharmacology and Therapeutics College of Medicine and Health Sciences, United Arab Emirates University Al Ain UAE; ^5^ Department of Pharmaceutical Chemistry, Faculty of Pharmacy Mutah University Al‐Karak Jordan

**Keywords:** awareness of skin disorders, hyperpigmentation, pregnancy‐related skin changes, preventive skincare, products for skincare

## Abstract

**Background:**

Hormonal, metabolic, and immunologic factors may cause several skin changes during pregnancy. Therefore, it is important for pregnant women to be aware of these expected changes in their skin appearance in order to prevent unwanted effects and to choose the appropriate preventive or treatment measures via trustable sources of information.

**Objective:**

This study would highlight the most common normal and abnormal physiological skin changes mothers usually complain about before or after pregnancy. The most commonly used skin care products, their sources of knowledge, information, perception, and experiences on these problems and products were also considered. In addition, patient satisfaction levels and their sources and types of knowledge were also investigated.

**Methods:**

Across sectional survey was distributed among women who met the inclusion criteria and were citizens in the Hashimate Kingdom of Jordan. This survey was composed of a number of questions used for investigating participant's socio‐demographic characteristics, during and post‐pregnancy characteristics and medications used, comparison of skin related complains reported by these women, in addition to their use of skin care products. Women's knowledge, perception, and experience regarding their use of skin care products were also included.

**Results:**

Of the 337 participants in this study, 6.5% and 6.8% of women were using thyroid medications and antihypertensive agents. An increase of around 3% in pregnancy‐related skin changes, such as hyperpigmentation, hair loss, cellulite, and wrinkles, was predominant among women. Despite the prevalence of hyperpigmentation, only 4% and 17% of women used depigmentation and sunblock products. Moreover, women declare that their information about the use of skin care products was mainly via social media or self‐experience. A low rate of consultation with dermatologists and the reliance on self‐diagnosis or non‐professional advice were shown.

**Conclusion:**

This study suggests a lack of awareness about effective preventive measures for skin‐related disorders commonly occurring during pregnancy, potentially exacerbated by reliance on unverified sources of information, such as social media. Therefore, incorporating education about skin changes into routine prenatal care could empower women to make informed decisions and reduce the stigma associated with these changes.

## 1 Introduction

Pregnancy is a special physiological condition characterized by numerous hormonal, metabolic, and immunologic changes that support the development of the fetus [1]. These changes often lead to noticeable alterations in a woman's body, particularly in the skin [2]. While most of these changes are transient, their appearance often leads to psychological concerns among pregnant women. Cultural, societal, and individual expectations regarding beauty and skin health are the leading causes of such concerns that may prompt some women to seek interventions and advice based on nonscientific grounds [3].

During pregnancy, skin hyperpigmentation, stretch marks, and vascular changes are commonly experienced by women caused by significant hormonal changes that may affect skin appearance in different ways [4, 5, 6]. Although these changes are generally harmless and resolve postpartum, their visibility can distress expectant mothers. Misconceptions that these changes indicate underlying health issues or permanent damage may push these women to choose unproven treatments [7, 8, 9].

Cultural perceptions of beauty and skin health play a very important role in the pregnant woman's perception and response to these commonly occurring changes [10]. In most cultures, good health is associated with clear, unblemished skin, which in turn suggests attractiveness and desirable social status. Hence, the changes brought on by pregnancy, such as melasma or stretch marks, may be perceived as undesirable or stigmatizing. This may force women to look for ways of restoring their skin appearance, using sometimes uncontrolled or unsafe products [11, 12, 13].

Skincare companies have capitalized on these concerns by marketing a wide array of products for the prevention and treatment of pregnancy‐related skin changes from topical creams and oils to prevent stretch marks, to depigmenting agents such as topical retinoids and hydroquinone [14]. While some products are based on clinical evidence, many lack rigorous testing for safety and efficacy during pregnancy [15, 16, 17].

Most women are dissatisfied with over‐the‐counter products or home remedies for pregnancy‐related skin changes due to unrealistic expectations or a lack of visible improvement [18, 19]. The reasons for dissatisfaction with these products are usually related to the intrinsic limitations of topical treatments in addressing conditions such as pigmentation and melasma, and due to the complex hormonal and genetic factors involved in the development of these conditions [20, 21]. Additionally, unrealistic expectations created by advertising these products create expectations that are far more than what is possible to be delivered, which also adds to the frustration [22]. Moreover, the limited awareness and knowledge of what constitutes a normal change and what does not, and the potential risks associated with some treatments used based on non‐professional advisors such as social media influencers and others [23].

Therefore, understanding the perceptions and satisfaction of women with the available skin care products, which are used to reduce the appearances of skin changes caused by pregnancy, is critical for implementing effective and safe interventions. This study focuses on investigating common physiologic and pathological skin changes associated with pregnancy and post‐partum stages, womens' perception about such changes, sources of information and experience with use of various skin care products. By identifying gaps in knowledge and investigating patient satisfaction, this study aims to provide insights into how healthcare providers can better support pregnant women to prevent large changes in their skin appearance. Additionally, it will examine the influence of cultural and societal factors on women's choices and perceptions regarding skin changes during pregnancy.

## 2 Methods

### 2.1 Sample Size

The sample size used in this study was calculated online using an online sample calculator (Raosoft). Based on the statistical data and official figures released by the Department of Statistics of the Hashemite Kingdom of Jordan titled: Population and Family Health Survey (2017–2018) [24], the population of the country is about 10 459 865. Therefore, 400 participants were needed to obtain a 95% confidence interval and a 5% margin of error. After exclusion of incomplete and contradictory responses, the final data included 388 participants.

### 2.2 Study Instrument

A cross‐sectional survey was conducted between May 31, 2024 and July 1, 2024. Women included in the study were informed of the main aims of the study and what it would entail by a professional dermatologist and pharmacists taking part in the study. An anonymous, electronic, self‐administered questionnaire was distributed and completed by participants who met the inclusion criteria.

### 2.3 Inclusion and Exclusion Criteria

Women ≥ 18 years old, with previous pregnancy(s), and living in the Hashimate Kingdom of Jordan were enrolled in the study.

Women who had not been previously pregnant, not living in Jordan, and younger than 18 years were excluded.

### 2.4 Questionnaire Survey

The questionnaire was designed and written in English, based on published literature, and was distributed to the public using an online survey portal and Google forms.

Data was collected by a previously validated questionnaire, developed by the research group and revised by experts in the field. Questions were distributed into four parts based on their type. Part 1: Women's socio‐demographic characteristics. Part 2: Women's characteristics and medications used during and post‐pregnancy. Part 3: Comparison of skin‐related complaints by women: before and during/after pregnancy, and their choices of use of skin care products. Part 4: Women's attitude, knowledge, behavior, perception, and experience regarding their use of skin care products during/after pregnancy.

### 2.5 Ethics Approval

An informed consent form was included at the beginning of the survey in the form of the question: Do you want to participate in this survey? and the statement: It is voluntary, and not obligatory to participate; you are free to withdraw at any time you want. The study objectives and their importance were described and explained to participants through written information in the survey.

This study was conducted in accordance with the Declaration of Helsinki [14]. Institutional approval was obtained from the scientific research and ethics committee—faculty of pharmacy at Mutah university, Al‐Karak, Jordan with code number: SREC 2024‐2025/4.

### 2.6 Statistical Analysis

The responses were analyzed using Statistical Package for Social Sciences (SPSS; IBM Corp., Armonk, NY, USA) version 22.0 and Excel program. Data were tabulated as percentages and frequencies for descriptive statistics. The possible relationships among different variables were assessed using the chi‐squared test. Differences were assumed to be significant at (*p* < 0.05).

## 3 Results

### 3.1 Women's Socio‐Demographic Characteristics

Table 1 shows that the age of majority (31.2%) of women in our sample was > 40 years, and (40.9%) of them already had ≥ 5 pregnancies; (51.4%) were smokers. Most women (85.8%) were living in Amman (the capital city), had attended university education level (74.2%), and of these (41.3%) were mainly professionals in the medical, allied medical, pharmaceutical, or cosmetologically field. Of the study participants, (58.8%) were employed, with (27.6%) having a middle income range of (500–1000 JD).

**TABLE 1:** Socio‐demographic characteristics of women (*n* = 337).

Characteristics	Frequency (*n*)	(%)
Age (years)		
18–19	7	2.1
20–25	29	8.6
26–30	56	16.6
31–35	76	22.6
36–40	64	19
> 40	105	31.2
Number of previous pregnancies		
1	1	3.6
2	25	7.4
3	62	18.4
4	100	29.7
5–7	115	34.1
> 7	23	6.8
Place of residency		
Albalqa	2	0.6
Zarqa	33	9.8
Ajloon	1	0.3
Amman	289	85.8
Aqpa	1	0.3
Irbid	8	2.4
Karak	3	0.9
Educational level		
High school	87	25.8
Graduate	199	59.1
Postgraduate	51	15.1
Educational sector		
MD	12	3.6
Pharmacist	44	13.1
Medical allied science	64	19
Cosmetology	19	5.6
Others	111	32.9
High school degree	87	25.8
Occupation		
> 7 h/day	67	19.9
< 6 h/day	92	27.3
Self‐employed	39	11.6
Not working	139	41.2
Income (JOD/month)		
< 500	88	26.1
500–1000	93	27.6
> 1000	23	6.8
No income	133	39.5
Smoking status		
Cigarette	71	21.1
Shisha	102	30.3
None smoking	164	48.7

### 3.2 Women's Characteristics: During and Post‐Pregnancy

Table 2 revealed that (76.6%) of women had full‐term pregnancy, with (60.8%) of the participants having their last pregnancy ≤ 10 years ago. In addition, (40.1%) of them used to do breastfeeding for their newborn babies for the first year of birth. The majority (55.8%) were using multiple supplements including Ferrous, Multivitamins, Skin, hair, and nails supplements, Vitamin D, or Calcium during and/or after pregnancy.

**TABLE 2:** Pregnancy characteristics of women (*n* = 337).

Characteristics	Frequency (*n*)	(%)
Pregnancy duration		
Full term	258	76.6
Preterm	79	23.4
Last delivery date		
< 1 year	22	6.5
1–3 years	71	17.75
4–6 years	82	20.5
7–9 years	30	7.5
≥ 10 years	132	39.2
Breast feeding after delivery during the first year		
Yes	135	40.1
No	202	59.9
Supplements used during pregnancy period		
Multiple supplements^a^	223	55.8
Collagen	7	2.1
Folic acid	13	3.9
Multivitamins only	39	11.6
Skin, hair, and nails supplements	11	3.3
Herbal drink	14	4.2
Vitamin D only	21	6.2
Nothing	17	5

^a^ Ferrous, Multivitamins, Skin, hair, and nails supplements, Vitamin D, Calcium.

Table 3 revealed that only (25.5%) of women were using chronic medications during their pregnancies, including mainly thyroid and antihypertensive agents. Findings revealed that a significant correlation (*p* < 0.05) was observed between these women on chronic medications and those with full‐term pregnancies.

**TABLE 3:** Medical conditions and medications used during pregnancy (*n* = 337).

Characteristics	Frequency (*n*)	(%)
Medical conditions		
Chronic medications	86	25.5
Non‐chronic medications	251	74.5
Medications used for chronic medical conditions		
Hypoglycemia	15	4.5
Anti‐Hypertensive	23	6.8
Joint pain	3	0.9
Stomach pain	5	1.5
Thyroids	22	6.5

### 3.3 Comparison of Skin Related Complains by Women: Before and During/After Pregnancy

Of the 337 participants in this study, most of them reported complaints of skin problems (88.76%) before pregnancy, while the majority (91.12%) complained of skin‐related problems that appeared ring pregnancy.

**FIGURE 1:**
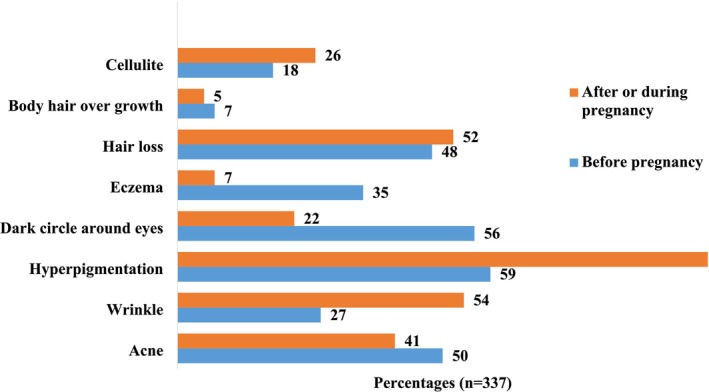
Comparison in percentages of skin‐related complaints by women: before and during/after pregnancy (*n* = 337).

Figure 1 showed all participated women (100%) complained of skin hyperpigmentation developed during/after pregnancy, compared to only (59%) of them complained before pregnancy of hyperpigmentation conditions. Similarly, cellulite, hair loss, and wrinkles complaints showed to increase in frequencies among participated women during/after pregnancy, compared to their frequencies before pregnancy.

On the contrary, body hair growth conditions, eczema, dark circles around eyes, and acne showed declining frequencies among women during and after pregnancy compared to their frequencies before pregnancy.

**FIGURE 2:**
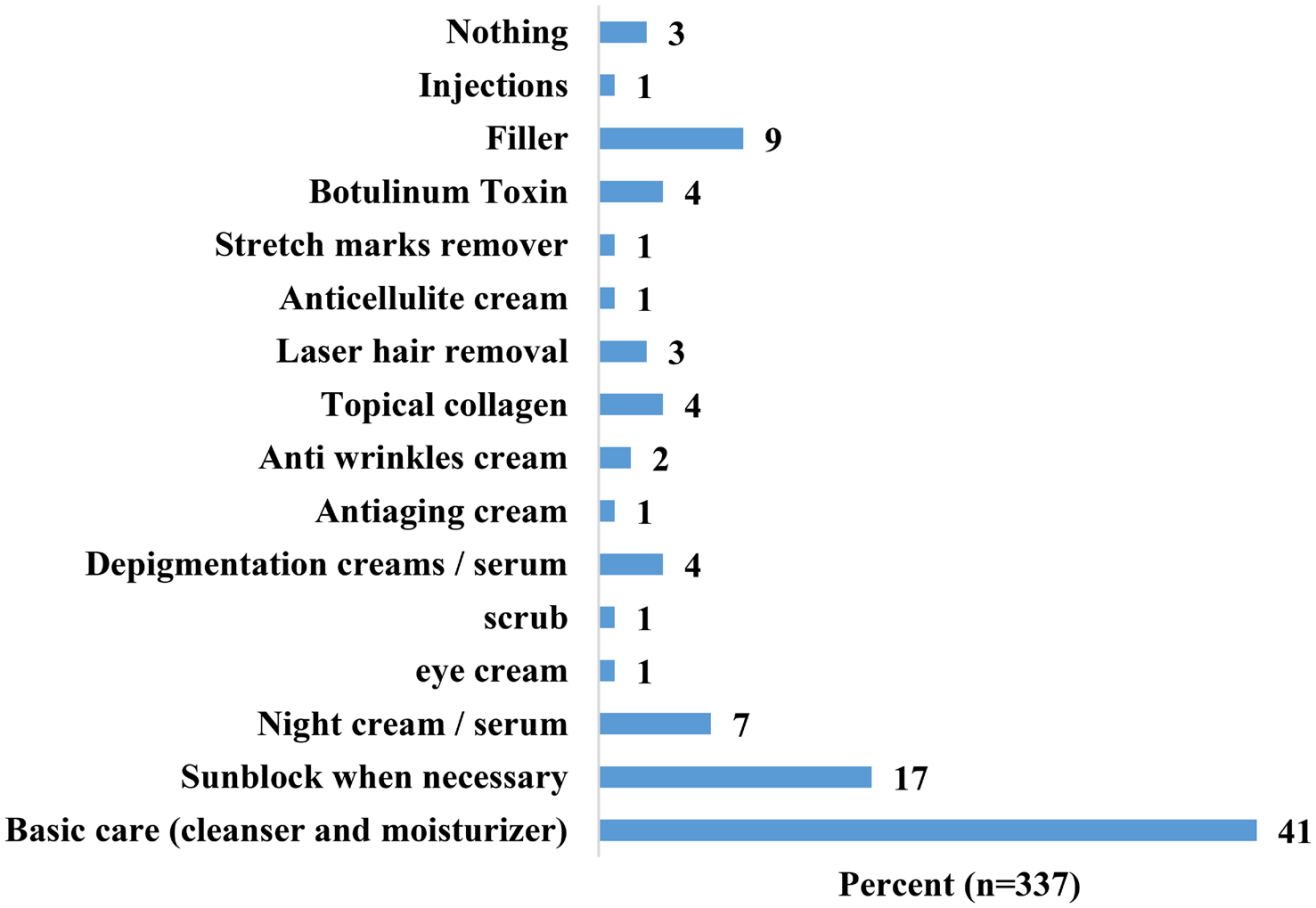
Percentages of skin care products used by women (*n* = 337).

Of the participants, approximately 41% reported using a basic skin care routine, including cleansers and moisturizers. Only 17% reported using sunblock. Despite the fact that more than half the women complained of skin hyperpigmentation, only 4% of them reported using depigmentation products (Figure 2).

### 3.4 Women's Attitude, Knowledge, Behavior, Perception, and Experience Regarding Their Use of Skin Care Products During/After Pregnancy

**FIGURE 3:**
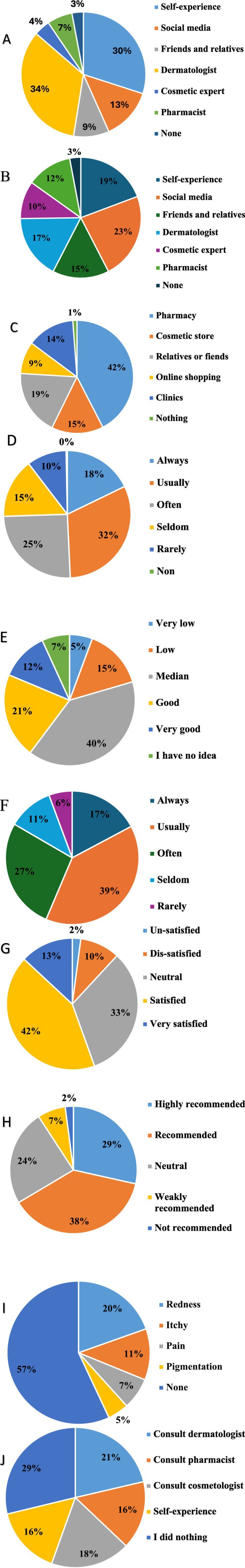
Women's attitude, knowledge, behavior, perception and experience regarding their use of skin care products during/after pregnancy (*n* = 337). (A) Method of diagnosis for skin conditions. (B) Source of information about women's choices of skin care products. (C) Proportion of sources used to buy skin care products. (D) Proportion of women reporting their regular use of skin care products. (E) Level of knowledge related to the use of skin care products. (F) Perception of women related to the benefit of using skin care products. (G) Level of women's satisfaction related to the use of skin care products. (H) Level of women's recommendation for others to use skin care products after pregnancy and lactation. (I) Side effects associated with the use of skin care products as reported by women. (J) Consultation perception for side effects associated with the use of skin care products as reported by women.

Figure 3A–J showed the methods of diagnosis, knowledge, experiences, beliefs, and perceptions about skin‐related conditions and products used among the participant women. Of the participants complaining of skin conditions, only (34%) were diagnosed by a dermatologist (Figure 3A). Others were diagnosed by themselves based on previous experience, social media, friends and relatives, or others. Significant correlations (*p* < 0.05) were observed between the different methods participants chose to diagnose their skin conditions with their occupation, income, and educational levels.

Findings also revealed that the main source of womens' information about the use of skin care products were social media, and secondly theirs' self‐experiences (Figure 3B). In addition, a proportion of (42%) among the participants revealed that they were buying their products from retail pharmacies (Figure 3C). Moreover, many of the participants reported that they were using skin care products on a usual or often frequency (32% and 25%, respectively) (Figure 3D).

Most of the participants (40%) showed a medium level of knowledge on using these products to treat different skin‐related problems (Figure 3E). Moreover, (39%) of the participants believed that the use of these treatments had usually helped them in reducing the symptoms of complaints (Figure 3F). Similarly, many of the participants felt satisfied regarding the results obtained using these products and recommended others to consider the use of skin care products after delivery and lactation, (42% and 38%) respectively (Figure 3G,H).

Results also showed that (43%) of the participants complained of varied side effects they experienced after using these products, which were mainly noted as skin redness (Figure 3I). While only (21%) consulted a dermatologist to help them manage their side effects, around one third of them (29%) did nothing regarding these side effects (Figure 3J).

## 4 Discussion

Up to our knowledge, this study is the first of its kind that sheds light on the diverse physiological and pathological skin changes that occur during and after pregnancy, as well as the perceptions, knowledge, and behaviors of women in response to these changes. Findings showed the reliance of the participants on non‐medical‐based advice, which highlights critical gaps detected among healthcare providers. Consequently, many women do not receive optimal recommendations for skin care routines during or after the pregnancy period, which hinders safe and efficient treatments and practices to be applied [18].

In this study, few participants reported being on thyroid medications. In a recent study in Jordan, findings showed that thyroid dysfunctions are common among female patients during pregnancy [25], which could be associated with skin complications. It is well known that thyroid dysfunctions, especially hypothyroidism and hyperthyroidism, often lead to worsening skin appearance [26]. Thyroid medications, such as levothyroxine, aim to normalize thyroid hormone levels, which can improve skin symptoms over time. However, improper dosing or nonadherence may exacerbate skin issues [27]. Hypothyroidism is commonly associated with dry, rough, and scaly skin due to reduced metabolic activity, which affects the skin's hydration and lipid production. Conversely, hyperthyroidism can cause smooth, moist, and thin skin due to increased metabolic activity and sweating [28]. This correlation underscores the need for close monitoring of thyroid function and adjustment of medication dosages during pregnancy and postpartum to minimize any thyroid‐related adverse effects on skin health. Therefore, healthcare providers are required to improve the communication strategies with these patients in order to reveal their use of such medications [29].

In this study, few of the participants reported using antihypertensive agents. This may reflect direct drug effects, or the vascular changes associated with hypertension itself, which can compromise skin integrity and healing [30]. Antihypertensive medications, commonly used to manage pregnancy‐induced hypertension or chronic hypertension, can also affect the skin, leading to side effects like redness and dryness in the skin, or exacerbation of conditions such as psoriasis due to their impact on vasodilation and immune modulation, in addition to their known effect as a photosensitizing agent [31].

It is well known that during pregnancy, the body undergoes a significant hormonal changing, particularly in estrogen, progesterone, and other pregnancy‐specific hormones which play a crucial role in maintaining pregnancy and preparing the body for childbirth [4]. Yet, they also have a profound impact on skin physiology leading to skin changes that include hyperpigmentation, and other changes [5]. Hyperpigmentation is well known as one of the most noticeable changes that occurs during pregnancy, often manifesting as linea nigra, darkened areolas, and melasma, commonly referred to as the “mask of pregnancy” [6]. As expected, in this study results revealed an increase in the frequency of appearance for different pregnancy‐related skin changes, such as hyperpigmentation, hair loss, cellulite, and wrinkles. All these changes were predominant among the participants comparing to their frequencies before pregnancy period. On the contrary, the occurrence of body hair over‐growth, eczema, eyes dark circles, and acne were less frequent after or during pregnancy periods.

Despite the prevalence of skin hyperpigmentation among the participants, only a few women used depigmentation products and were regularly using sunblock products. Moreover, findings revealed that many participants had medical or pharmaceutical education backgrounds, yet they relied on information gathered from social media for using skin care products. In this context, previous similar work revealed that pregnant women often rely on subjective advice via online forums or unverified sources when selecting skin care products, therefore increasing the risk of using inappropriate or harmful substances [16], specifically the depigmentation agents such as retinoids or hydroquinone, which are contraindicated during pregnancy [17]. These findings shed light on the need that even for women with medical education or professional backgrounds, it is still vital that they should be informed by their health care provider about the importance of enhancing their knowledge, as well as the need to rely on trusted sources of information regarding the use of skin care products during or after pregnancy periods. These trends also emphasize the need for promoting safe skin care practices during pregnancy and addressing beauty norms that perpetuate such behaviors.

Results also showed that participants tend to possess a low rate of consultation with dermatologists, and that they rely more often on self‐diagnosis or non‐professional advice when they need to use skin care products. In addition, many of the participants expressed a moderate level of knowledge on using skin care products to treat different skin‐related problems or complaints. Therefore, patient satisfaction and willingness to recommend skin care products to others were also low. These findings were expected, as dissatisfaction stemmed from unrealistic expectations as well as the side effects associated with the use of these products (e.g., skin redness). Previous studies on societal pressures in regions like South Asia and Africa have been shown to influence the use of skin‐lightening products, some of which contain harmful ingredients, such as mercury and high‐dose corticosteroids, which pose serious health risks, including potential toxicity and teratogenic effects [12, 13].

Previous study by Shroukh et al. [32] agrees with the current findings, as it revealed that healthcare providers can play a pivotal role in bridging the knowledge gap among patients by additional training, which focuses on the importance of raising awareness about different health changes including pregnancy. Therefore, the findings of this study suggest that better communication and discussing skin changes with pregnant women, aiming to offer medical‐based advice, may help to prevent the misuse of treatments. Moreover, results emphasize the need for healthcare providers and the skin care industry to set realistic expectations and prioritize the safety and efficacy of products tailored for pregnant and postpartum women.

In conclusion, this study highlights significant gaps in knowledge, behavior, and perceptions related to pregnancy‐related skin changes. Addressing these gaps through education, evidence‐based product recommendations, and culturally sensitive interventions is critical. By fostering better communication between healthcare providers and these patients, we can improve the outcomes as well as satisfaction while reducing unnecessary or harmful practices.

## Author Contributions

Safwan Aladwan, Reem Issa, and Lidia Kamal Al‐Halaseh: conceptualization, project coordination. Safwan Aladwan, Reem Issa, and Wala'a Al. Safadi: writing introduction and the discussion, development of the first draft of the manuscript. Safwan Aladwan and Reem Issa: writing the methodology and the study design. Safwan Aladwan, Reem Issa, and Wala'a Al. Safadi: data collection. Reem Issa and Lidia Kamal Al‐Halaseh: preparing the tables and figures. Lilian Alnsour and Safwan Aladwan: contributing to data analysis. Wala'a Al. Safadi, and Lidia Kamal Al‐Halaseh: final proofreading before submission.

## Ethics Statement

Ethical approval for the study was obtained by the Scientific Research and Ethics Committee at the Faculty of Pharmacy, Mutah University, Al‐Karak, Jordan. This was obtained by adhering to the Declaration of Helsinki's ethical standards for human research, the International Conference on Harmonization's Good Clinical Practice Guideline, and the NMPA's Guideline for Good Clinical Practice.

## Conflicts of Interest

The authors declare no conflicts of interest.

## Supporting information


Data S1.

## Data Availability

The data that supports the findings of this study are available in the Supporting Information of this article.
